# Anti-Bacterial Activity of Recombinant Human β-Defensin-3 Secreted in the Milk of Transgenic Goats Produced by Somatic Cell Nuclear Transfer

**DOI:** 10.1371/journal.pone.0065379

**Published:** 2013-06-14

**Authors:** Jun Liu, Yan Luo, Hengtao Ge, Chengquan Han, Hui Zhang, Yongsheng Wang, Jianmin Su, Fusheng Quan, Mingqing Gao, Yong Zhang

**Affiliations:** College of Veterinary Medicine, Northwest A&F University, Key Laboratory of Animal Biotechnology of the Ministry of Agriculture, Yangling, Shaanxi, China; University of South Florida College of Medicine, United States of America

## Abstract

The present study was conducted to determine whether recombinant human β-defensin-3 (rHBD3) in the milk of transgenic goats has an anti-bacterial activity against *Escherichia coli (E. coli)*, *Staphylococcus aureus (S. aureus)* and *Streptococcus agalactiae (S. agalactiae)* that could cause mastitis. A HBD3 mammary-specific expression vector was transfected by electroporation into goat fetal fibroblasts which were used to produce fourteen healthy transgenic goats by somatic cell nuclear transfer. The expression level of rHBD3 in the milk of the six transgenic goats ranged from 98 to 121 µg/ml at 15 days of lactation, and was maintained at 90–111 µg/ml during the following 2 months. Milk samples from transgenic goats showed an obvious inhibitory activity against *E. coli*, *S. aureus* and *S. agalactiae in vitro*. The minimal inhibitory concentrations of rHBD3 in milk against *E. coli, S. aureus* and *S. agalactiae* were 9.5–10.5, 21.8–23.0 and 17.3–18.5 µg/mL, respectively, which was similar to those of the HBD3 standard (*P>*0.05). The *in vivo* anti-bacterial activities of rHBD3 in milk were examined by intramammary infusion of viable bacterial inoculums. We observed that 9/10 and 8/10 glands of non-transgenic goats infused with *S. aureus* and *E. coli* became infected. The mean numbers of viable bacteria went up to 2.9×10^3^ and 95.4×10^3^ CFU/ml at 48 h after infusion, respectively; the mean somatic cell counts (SCC) in infected glands reached up to 260.4×10^5^ and 622.2×10^5^ cells/ml, which were significantly higher than the SCC in uninfected goat glands. In contrast, no bacteria was presented in glands of transgenic goats and PBS-infused controls, and the SSC did not significantly change throughout the period. Moreover, the compositions and protein profiles of milk from transgenic and non-transgenic goats were identical. The present study demonstrated that HBD3 were an effective anti-bacterial protein to enhance the mastitis resistance of dairy animals.

## Introduction

Mastitis is inflammation of the mammary gland, which is usually caused by microbial infection [1]. Bovine mastitis is highly prevalent and the most costly disease in the dairy industry worldwide [2]. Traditional antibiotic treatments for clinical mastitis have resulted in antibiotic-resistant bacterial strains and antibiotic residues in milk [3]. In addition, there is no effective vaccine for the management of mastitis [4]. Recently, several research teams have demonstrated that the expression of anti-bacterial proteins in the milk of transgenic dairy animals can inhibit the bacterial pathogens that cause mastitis [5]–[8]. Therefore, the production of mastitis-resistant animals by genetic engineering technology has been proposed as an alternative approach to enhance mastitis resistance [9].

It has been reported that some kinds of anti-bacterial peptides (AMPs) play an important role in the innate immunity of the bovine mammary gland [10]. Beta-defensins, a kind of AMPs, are expressed in response to mastitis, and protect the body from bacterial invasion [11], [12]. Scientists have extensively studied human β-defensin-3 (HBD3). HBD3 is widely expressed in many tissues [13], [14], has a broad-spectrum anti-bacterial activity against bacteria, fungi and enveloped viruses, and plays important roles in immunity [15]. Therefore, HBD3 may be a candidate gene to enhance mastitis resistance. In addition, recombinant HBD3 (rHBD3) purified from milk may be useful for pharmacological and therapeutic applications.

The current study was designed to determine whether rHBD3 secreted in the milk of cloned transgenic goats has an anti-bacterial activity against bacteria that could cause mastitis. We transfected a HBD3 mammary-specific expression vector into goat fetal fibroblast cells (GFFs) by electroporation, and produced transgenic goats by somatic cell nuclear transfer (SCNT). In addition, we examined the *in vitro* and *in vivo* anti-bacterial activity of rHBD3 in the milk of cloned transgenic goats. This study demonstrated that HBD3 could be an effective anti-bacterial protein to enhance the mastitis resistance of dairy animals.

## Materials and Methods

### Ethics Statement

All experiments were approved by Care and Use of Animals Center, Northwest A&F University. This study was carried out in strict accordance with the Guidelines for the Care and Use of Animals of Northwest A&F University. Goat’s ovaries were collected from Tumen abattoir, a local slaughterhouse of Xi’An, P.R. China. Fetuses and recipient goats were obtained from Yangling Keyuan Cloning Co., Ltd. Every effort was made to minimize animal pain, suffering and distress and to reduce the number of animal used, and all surgery was performed under anesthesia created by intravenous injection of Sumianxing (Veterinary Research Institute, Jilin, China).

### Chemicals

Unless otherwise indicated, all chemicals and reagents were purchased from Sigma Chemical Company (St. Louis, MO), and the culture medium and fetal bovine serum (FBS) used for preparation of donor cells were obtained from Gibco (Grand Island, NY).

### Construction of the HBD3 Mammary-specific Expression Vector pEBB

**Figure 1:**
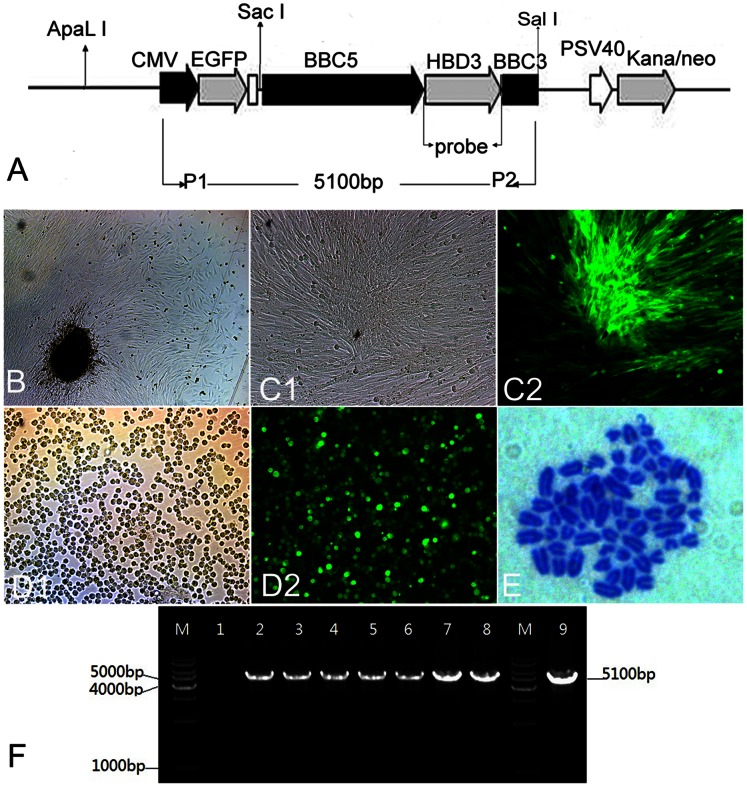
Generation of transgenic donor cells. (A) Schematic representation of the recombinant plasmid pEBB. Positions of primers (P1/P2) and the probe used for PCR and Southern blot are showed. (B) Primary culture of GFFs. (C) A G418-resistant colony expressing EGFP under bright field (C1) and fluorescence (C2). (D) Transgenic cells expressing EGFP under bright field (D1) and fluorescence (D2) digested by a 0.25% trypsin solution. (E) Chromosome number in transgenic cells (2n = 60). (F) PCR analysis of G418-resistant colonies. M, Marker; Lane 1, untransfected GFFs (negative control); Lanes 2–8, G418-resistant colonies; Lanes 9, pEBB vector (positive control).

Construction and assessment strategies of the mammary-specific expression vector pEBB were performed as described previously [16]. Genomic DNA was extracted from the blood of Holstein cattle using a TIANamp Genomic DNA Kit (Tiangen Biotech, Beijing, China). The 2.2 kb promoter region (BBC5, including the 1.7 kb 5′-flanking sequence, exon 1 and part of intron 1) and 0.6 kb 3′-untranslated region (BBC3, including part of the last intron and exon) of the bovine β-casein gene (GenBank: X14711) were amplified using rTaq DNA polymerase (Takara, Dalian, China). We obtained the 1156 bp HBD3 DNA sequence (GenBank: 55894) from human genomic DNA by PCR. Then, the pEBB vector was constructed by inserting 2.2 kb BBC5, 0.6 kb BBC3, and 1156 bp HBD3 DNA sequences into a pEGFP-C1 plasmid (Clontech, Mountain View, CA, USA) (Fig. 1A). The vector was linearized with *ApaL*I, purified by a Wizard DNA Clean-Up System (Promega, Madison, USA), diluted with double distilled water, quantified, and then used for transfection.

### Preparation of HBD3 Transgenic Donor Cells

GFFs were isolated as described previously [17]. An established cell line, GFF1, was seeded on a 60-mm culture dish, and cultured in 4 ml Dulbecco’s modified Eagle’s medium/Ham’s F-12 (DMEM/F12) supplemented with 10% FBS. At 70–80% confluence, cells were harvested and transfected by electroporation [18]. Briefly, the cell concentration was adjusted to 5×10^6^ cells/ml in transfection medium containing 20 µg/ml plasmid DNA. Then, the mixture was transferred to a 4-mm gap electroporation cuvette and pulsed by a BTX ECM 2001 (500 V, 1 ms, three pulses). After the pulses, cells were seeded on a 100-mm culture dish containing 10 ml DMEM/F12 supplemented with 10% FBS. After 24 h of culture, the cells were selected by 800 µg/ml geneticin (G418) for 7 days, and then 400 µg/ml G418 was used to obtain cell colonies. Cell colonies were counted under an inverted fluorescence microscope (Nikon, Tokyo, Japan). Enhanced green fluorescent protein (EGFP)-positive colonies were picked up and expanded in culture. A portion of the cell culture was harvested for cryopreservation, and the remaining cells were used for PCR screening. The number of chromosomes in transgenic clonal cells was determined as described previously [19]. More than twenty metaphase chromosome spreads from each sample were examined, and cells with 60 chromosomes were classified as normal cells. Each cell line was analyzed three times.

### Detection of Transfected Cells by PCR

G418-resistant clonal cells were lysed in lysis buffer, and then analyzed by PCR to confirm transgene integration. The upstream primer (5′-GGTCATTAGTTCATAGCCCATATATGGAGTTC-3′) is located in the CMV promoter, and the downstream primer (5′-CGTCGACTTTCCACAGCTCTTTTTAACATC3-′) is located in BBC3 (Fig. 1A, P1/P2). LATaq DNA Polymerase (Takara, Dalian, China) was used to amplify a 5100 bp fragment containing CMV promoter, EGFP, BBC5 and BBC3 sequences. PCR amplification conditions were 94°C for 5 min, followed by 30 cycles of 94°C for 30 sec, 58°C for 30 sec and 72°C for 4 min, and then 72°C for 10 min. Following amplification, PCR products were analyzed on a 0.8% agarose gel containing ethidium bromide. PCR products isolated from the gel were cloned into a pMD19-T vector using a TA Cloning Kit (Takara, Dalian, China), and then sequenced. PCR analysis was also performed on the primary cultured fibroblasts (GFF1) and pEBB plasmid as negative and positive controls, respectively.

### Somatic Cell Nuclear Transfer

Transgenic cells were seeded on a 48-well plate, and allowed to reach confluence for 2–3 d prior to SCNT. The SCNT procedures were performed as described previously by our laboratory [20], [21]. Briefly, oocytes with the first polar body were selected for enucleation after 22–24 h of *in vitro* maturation. Both the polar body and metaphase plate were removed, and then a single round donor cell was injected into the perivitelline space of the enucleated oocyte. Karyoplast-cytoplast couplets were fused by electrofusion. Couplets were incubated for 2–3 h in TCM-199 supplemented with 10% FBS and 7.5 µg/ml cytochalasin B. Fused embryos were activated by treatment with 5 µM ionomycin for 5 min and then 2 mM 6-dimethylaminopurine for 4 h. Following activation, the embryos were washed extensively and cultured in 200 µl mSOF supplemented with 10% FBS while covered with mineral oil at 38.5°C in a humified atmosphere with 5% CO_2_. Embryos were cultured for 7 days to evaluate the *in vitro* developmental rate. One- to two-cell stage embryos cultured for 20–24 h were transferred into the oviducts of synchronized recipients on day 1 of estrus (day 0 = estrus, 19–28 embryos per recipient). Pregnancy was determined by ultrasonography.

### Identification of Transgenic Goats by PCR and Southern Blotting

Genomic DNA from cloned goats was extracted, and PCR was performed as described above. Genomic DNA from wild-type goats and transgenic cells was used as negative and positive controls, respectively.

Genomic DNA isolated from cloned goats was analyzed by Southern blotting using a DIG-High Prime DNA Labeling and Detection Starter Kit II (Roche Molecular Biochemicals, Mannheim, Germany). Genomic DNA (20 µg) was thoroughly digested with restriction endonucleases *Sac*I and *Sal*I (New England Biolabs, Beverly, MA). Digested DNA was separated by 1.0% agarose gel electrophoresis, and then transferred to nylon membranes (Amersham, Piscataway, NJ). A digoxigenin-labeled 1156 bp HBD3 DNA probe (Fig. 1A, Probe) was prepared following the manufacturer’s recommended procedure. The membranes were subsequently hybridized with the probe overnight at 68°C in a hybridization solution. The bands were detected using a chemiluminescent substrate system.

### Milk Collection and Composition Analysis

At about 16 months of age, transgenic goats were naturally mated, and pregnancy was detected by ultrasonography at around 60 days after fertilization. After delivery, milk samples were collected from transgenic and non-transgenic goats (negative control) of the same breed and age. The content of fat, protein, lactose, and dry matter was determined using a MilkoScan FT-120 (Foss, Hillerod, Denmark). The general profile of milk proteins was examined by Tricine-sodium dodecyl sulfate-polyacrylamide gel electrophoresis (Tricine-SDS-PAGE) and Coomassie Blue staining. Secretion of HBD3 in the milk of transgenic goats was analyzed according to the following procedures.

### Tricine-SDS-PAGE and Western Blot Analysis of Transgenic Milk

Secretion of HBD3 in the milk of transgenic goats was analyzed by Tricine-SDS-PAGE and western blot analysis. Milk samples were centrifuged at 3,000 *g* for 15 min to remove the fat fractions. Liquids from the lower layer were then adjusted to pH 3.8–4.6 with 1 M HCl to eliminate the casein fraction. Then, 5 µl milk samples were mixed with the same volume of loading buffer and separated by 12% Tricine-SDS-PAGE. After electrophoresis, proteins were stained with Coomassie Brilliant Blue to examine the protein composition in the milk, or were transferred onto polyvinylidene difluoride membranes in a Bio-Rad trans-blot Cell (Bio-Rad, Hercules, CA, USA). After blocking, the membranes were reacted with a rabbit anti-HBD3 antibody (Sigma) at a 1∶100 dilution, and then incubated with an alkaline phosphatase-conjugated goat anti-rabbit IgG (Beyotime Institute of Biotechnology, Shanghai, China) at a 1∶1000 dilution. The membranes were washed extensively and exposed to Kodak XBT-1 film in a dark room. Milk from non-transgenic goats was used as a negative control, and 10 µg HBD3 standard (Premedical, Beijing, China) was used as a positive control. In addition, whole milk samples were analyzed by SDS-PAGE and western blotting using a rabbit anti-β-casein antibody (Ricky, Shanghai, China) to detect the expression of β-casein protein.

### Concentration Measurement of HBD3 in Milk

Concentrations of HBD3 in transgenic milk were determined by a sandwich enzyme-linked immunosorbent assay (ELISA) kit (Adipo Bioscience, Santa Clara, CA, USA) according to the manufacturer’s instructions. All samples were tested in duplicate, and the procedure was repeated three times.

### Analysis of HBD3 Anti-bacterial Activity *in vitro*

The anti-bacterial activity of rHBD3 in the milk of transgenic goats, non-transgenic controls, and non-transgenic control milk containing the HBD3 standard was characterized by two methods as follows. Three classes of microorganisms, *Escherichia coli* (*E. coli*, ATCC25922), *Staphylococcus aureus* (*S. aureus*, ATCC25923) and *Streptococcus agalactiae* (*S. agalactiae*, ATCC12386), were obtained from the Chinese Institute of Veterinary Drug Control. The microorganisms were grown overnight at 37°C in 50 ml trypticase soy broth medium. Bacterial suspension was centrifuged at 3,000 *g* for 5 min, and the bacteria were washed and resuspended in phosphate buffered saline (PBS) to 1×10^7^ colony forming units (CFU)/ml.

The anti-bacterial activity of transgenic milk was roughly estimated by inhibition zone assay. A bacterial suspension (100 µl) was spread onto a 100-mm agar dish and air dried for 10 min. Then, milk samples (20 µl) were spotted onto individual discs of quantitative filter paper (7 mm in diameter), which were placed on the agar dish containing *E. coli, S. aureus* or *S. agalactiae*. After incubation at 37°C for 24 h, inhibition zones around the filter paper discs indicated the anti-bacterial activity. Non-transgenic milk was spotted on the filter paper as negative controls, and non-transgenic control milk containing 100 or 200 µg/ml HBD3 standard was used as positive controls.

The minimal inhibitory concentration (MIC) of rHBD3 in milk from transgenic goats and the HBD3 standard was measured by a liquid growth inhibition assay as described previously [22] with slight modifications. Transgenic milk and the HBD3 standard were diluted in non-transgenic control milk at various final concentrations. Then, ten microliter bacterial suspension was added to 90 µl milk samples (1×10^5^ CFU/ml), followed by incubation for 3 h at 37°C. The samples were then diluted serially in PBS, plated on agar dish, and incubated for 18 h at 37°C. The number of colonies was then counted. The MIC is the lowest concentration at which 99.9% of the viable cells are inhibited. All assays were conducted in duplicate and repeated four times.

### *In vivo* Anti-bacterial Activity Assay

Mastitis resistance ability of HBD3 transgenic goats was examined by intramammary infusion of viable bacterial inoculums as described previously [7]. Six transgenic and 13 non-transgenic goats were used in this experiment. Before treatment (0 h), milk samples were collected from each gland, and the number of bacteria and somatic cell count (SCC) were determined to assess health of the animals. After milking, the right and left glands of each goats were infused with 1 ml *S. aureus* and *E. coli* inoculums (10^3^ CUF/ml in PBS), respectively, via the streak canal. Six glands of three non-transgenic goats received 1 ml sterile PBS. At 12, 24 and 48 h after infusion, milk samples were collected and plated on agar dish to count the colony forming unit. The SSC of milk samples was determined on Fossomatic FC instrument (Foss, Hillerod, Denmark) according to the manufacturer’s instructions. Glands containing fewer than 10 CFU/ml bacteria in milk were considered as not infected. After experiment, antibiotic therapy was administered to the infected goats.

### Statistical Analysis

All data were analyzed using SPSS 16.0 statistical software (IBM Corporation, Somers, NY). The *in vivo* developmental rates of cloned embryos were tested by Chi-square analysis. Data of *in vitro* developmental rates and anti-bacterial activities were represented as the mean±SEM, and results were analyzed by one-way ANOVA and least-significant difference tests. For all analyses, *P*<0.05 was considered significant.

## Results

### Preparation of HBD3 Transgenic Donor Cells

Nine GFF cell lines were established from different fetuses. The primary GFF1 cell line from a female fetus was used in this study (Fig. 1B). About 3×10^6^ cells were transfected with pEBB by electroporation. A total of 274 G418-resistant colonies were obtained, and 31 (14.4%) colonies expressed EGFP (Fig. 1C). We picked up 20 EGFP-positive colonies and transferred them to 48-well plates. Seven (35.0%) selected colonies were expanded to more than 2×10^6^ cells, and all cell populations were positive for fluorescence (Fig. 1D). After expansion in culture, a portion of the clonal cells was frozen or used as donor cells for SCNT, and the remaining cells were passaged routinely to determine the chromosome number (Fig. 1E) and integration of the transgene. The percentage of cells with a normal chromosomal number (2n = 60) in G418-resistant colonies (F1HBDC1, F1HBDC2, F1HBDC3, F1HBDC4, F1HBDC5, F1HBDC6, and F1HBDC7) were 72.3±1.2(50/69), 71.1±2.0(44/62), 59.9±1.4(42/70), 49.3±1.6(32/65), 51.5±0.8(34/65), 57.9±1.8(40/69), and 51.6±1.9(35/68) %, respectively. PCR amplification indicated presence of the transgene in the seven transgenic cell lines (Fig. 1F).

### Generation of Cloned Transgenic Goats by SCNT

**Figure 2:**
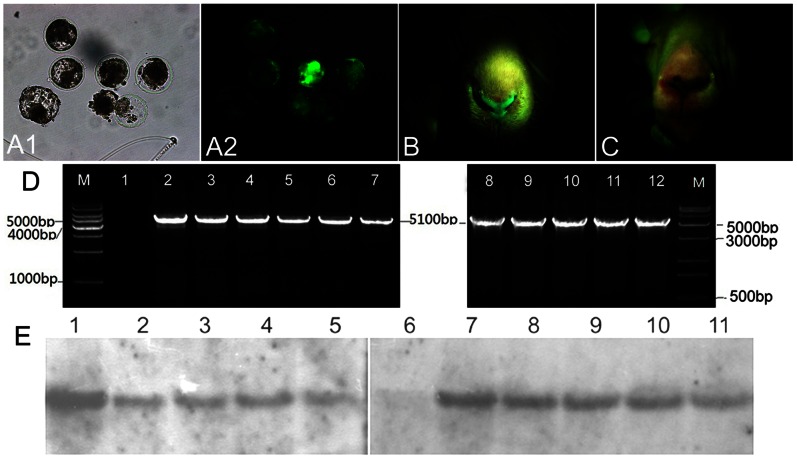
Identification of transgenic cloned goats. (A) Cloned transgenic blastocysts under bright field (A1) and fluorescence (A2). (B and C) EGFP expression in cloned goats was observed using a Dual Fluorescent Protein Flashlight (B), and no EGFP expression was observed in wild-type goats (C). (D) PCR analysis. M, Marker; Lane 1, wild-type goat (negative control); Lanes 2–3: transgenic cell lines F1HBDC1 and F1HBDC2 (positive control); Lanes 4–12: transgenic goats. (E) Southern blot analysis. Lane 1, pEBB vector (positive control); Lane 6, wild-type goat (negative control); Lanes 2–5 and 7–11, transgenic goats.

Most of the cloned blastocysts from all the transgenic cell lines expressed EGFP (Fig. 2A), but the *in vitro* developmental rates of cloned embryos from F1HBDC1 and F1HBDC2 were significantly higher than those from F1HBDC4, F1HBDC5, F1HBDC6, and F1HBDC7 (*P*<0.05, Table 1). After comprehensive examination, transgenic cell lines F1HBDC1 and F1HBDC2 were used as donor cells to produce cloned transgenic goats. A total of 1817 early-stage embryos were transferred into the oviducts of 79 recipient goats (Table 2). No significant difference was observed in pregnancy rates between these two cell lines at 30 days of gestation. However, the delivery rate of cell line F1HBDC2 was higher than that of F1HBDC1 (23.5% vs. 9.1%, *P*<0.05). The cloning efficiencies (offspring produced per embryo transferred) of F1HBDC1 and F1HBDC2 were 0.4% and 1.6%, respectively.

**Table 1:** *In vitro* development of cloned embryos from different transgenic cell lines.

Donor cells	Fusion rate*	Cleavage rate**	Blastocyst rate***
F1HBDC1	(141/162)87.0±1.1^a^	(105)74.6±0.9^a^	(31)22.0±0.7^a^
F1HBDC2	(134/161)83.2±0.6^a^	(100)74.8±1.5^a^	(30)22.3±1.1^a^
F1HBDC3	(137/158)86.8±0.9^a^	(103)75.2±1.6^a^	(29)21.2±0.4^ab^
F1HBDC4	(144/167)86.3±1.3^a^	(94)65.3±1.5^b^	(23)16.0±0.6^c^
F1HBDC5	(136/162)83.9±1.5^a^	(90)67.1±1.8^b^	(22)16.2±0.1^c^
F1HBDC6	(145/166)87.5±2.3^a^	(106)73.0±2.0^a^	(28)19.3±0.7^b^
F1HBDC7	(138/161)85.8±0.7^a^	(100)72.8±2.8^a^	(27)19.6±1.0^b^
GFF1	(142/167)85.1±2.4^a^	(107)75.5±1.3^a^	(31)21.8±0.6^a^

* fusion rate = No. of fused embryos/No. of couplets.

** cleavage rate = No. of cleavage embryos/No. of fused embryos.

*** blastocyst rate = No. of blastocyst/No. of fused embryos.

Four replicate experiments were performed per cells. Numbers in parentheses represent total embryo numbers of four replicates, while other numbers represent development rates (mean±SEM %).

a–c Within a column, values with different superscripts are significantly different from each other (*P*<0.05).

**Table 2:** *In vivo* development of cloned embryos from different transgenic cell lines.

Donor cell	No. of embryos cultured	No. of embryos transferred	No. of recipients	No. of pregnant (%)*		No. of kids born (%)**
				Day 30	Term	
F1HBDC1	279	253	11	4(36.4)^a^	1(9.1)^a^	1(0.4)
F1HBDC2	1632	1564	68	28(41.2)^a^	16(23.5)^b^	25(1.6)

* Pregnancy rate = No. of pregnant recipients/No. of recipients used.

** Cloning efficiency = No. of kids born/No. of embryos transferred.

a, b Within a column, values with different superscripts are significantly different from each other (*P*<0.05).

We obtained 26 cloned offspring, and the information on the cloned goats was summarized in Table 3. Gestation lengths ranged from 148 to 162 days, and birth weights ranged from 1.3 to 4.9 kg. Eighteen cloned goats were alive at birth, fourteen of which survived and no abnormality was observed. Mortality of the cloned goats was duo to placental defects, intrauterine infection, abnormal joints, or anal atresia, while no abnormalities were observed in some of the dead goats. The expression of EGFP in cloned goats was observed using a Dual Fluorescent Protein Flashlight (NightSea DFP-1, MA, USA), which revealed that the skin of cloned goats expressed a high level of EGFP (Fig. 2B and C). PCR detection indicated that all the cloned goats have integration of transgene HBD3 (date not shown). Nine transgenic goats grew to adulthood and were naturally mated, and the integration of transgene was detected by PCR using primers P1/P2 (Fig. 1A) and Southern blot using probe (Fig. 1A). Analysis of genomic DNA by PCR revealed integration of the transgene in cloned goats, whereas no transgene integration was detected in the surrogate female goat (Fig. 2D). Genomic Southern blot analysis further confirmed integration of the transgene in the genome of live offspring (Fig. 2E). After delivery, the nine transgenic goats were able to lactate normally. Six transgenic goats (TG3, TG7, TG10, TG14, TG15 and TG 18) at the same lactation stage (within 11 days) were used in the subsequent experiments.

**Table 3:** Summary of HBD3 transgenic goats.

Recipients	Cloned goats	Gestation length (day)	Birth weight (kg)		Postnatal status
55	TG1	151		2.7	stillborn
0340	TG2	152		2.0	normal
42	TG3	153		2.4	normal
42	TG4	153		1.7	normal
95	TG5	152		4.2	stillborn
54	TG6	162		3.1	stillborn
104	TG7	152		3.5	normal
03910	TG8	150		2.7	stillborn
03058	TG9	152		2.2	normal
87	TG10	151		3.5	normal
46	TG11	148		1.8	normal
46	TG12	148		2.1	normal
46	TG13	148		2.8	abnormal joints
0658	TG14	150		3.5	normal
0658	TG15	150		2.5	normal
52	TG16	154		1.9	spinal deformity
74	TG17	150		4.9	stillborn
100	TG18	151		3.3	abnormal joints
100	TG19	151		2.1	stillborn
018	TG20	151		2.8	stillborn
018	TG21	151		1.9	normal
0323	TG22	152		4.4	normal
124	TG23	151		2.0	normal
124	TG24	151		3.0	anal atresia
124	TG25	151		2.4	normal
124	TG26	151		1.3	stillborn

### Composition Analysis of Transgenic Milk

**Figure 3:**
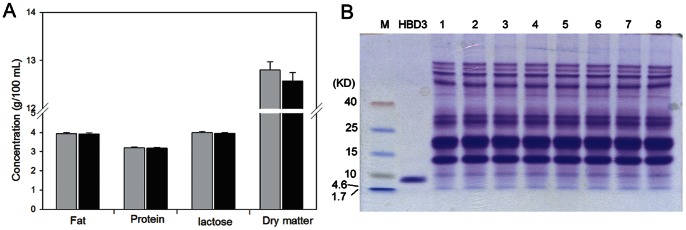
Composition analysis of transgenic milk. (A) Comparison of basic components between transgenic and non-transgenic milk. Gray bars, milk samples from transgenic goats (n = 6); black bars, milk samples from non-transgenic goats (n = 6). (B) Analysis of proteins in milk (excluding the casein fraction) by Tricine-SDS-PAGE and Coomassie Blue staining. Milk samples (5 µl) were loaded and analyzed. M, protein marker; HBD3, human β-defensin-3 standard; Lanes 1–2, milk samples from non-transgenic goats; Lanes 3–8, milk samples from transgenic goats.

Composition analysis of whole milk samples was performed on a MilkoScan FT-120 (Foss, Hillerod, Denmark). The content of total fat, protein, lactose and dry matter in milk samples obtained from transgenic goats (n = 6) was similar to those in milk samples from control goats (n = 6) (Fig. 3A). Furthermore, the total protein profiles of milk samples (excluding the casein fraction) were similar between transgenic and non-transgenic goats (Fig. 3B).

### Expression of HBD3 in the Milk of Transgenic Goats

**Figure 4:**
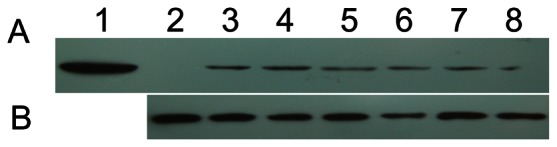
Expression of rHBD3 in transgenic goat milk. Protein expression of rHBD3 (A) and β-casein (B) were detected by western blotting. Lane 1, HBD3 standard; Lane 2, milk samples from non-transgenic goats; Lanes 3–8, milk samples from transgenic goats.

Milk samples were diluted and analyzed by western blotting. Milk from non-transgenic goats was used as a negative control, and 10 µg HBD3 standard was used as a positive control. Western blot analysis confirmed that rHBD3 (5 kD) was only present in the milk of transgenic goats (Fig. 4A). Beta-casein (30 kD) was present in the milk of both transgenic and non-transgenic goats, which was used as an internal control (Fig. 4B). These results indicated that rHBD3 was expressed in the milk of transgenic goats.

The concentration of rHBD3 in the milk of transgenic goats was further quantified by ELISA (Table 4). To evaluate the stability of rHBD3 expression, milk samples were collected at different stages of lactation. The results showed that the expression level of rHBD3 in milk of the six transgenic goats was from 98 to 121 µg/ml at 15 days of lactation, and maintained 90 to 111 µg/ml during the following 2 months.

**Table 4:** Concentrations of HBD3 in milk from transgenic goats.

Transgenic goats	Concentration of HBD3 (µg/ml)		
	15 days of lactation	45 days of lactation	65 days of lactation
TG3	101.2±0.6	90.7±0.2	89.7±0.6
TG7	120.5±0.1	110.8±0.2	110.8±0.7
TG10	116.4±0.5	104.0±0.5	104.9±0.2
TG14	98.4±0.4	90.7±0.2	90.3±0.2
TG15	116.5±0.4	110.4±0.2	111.8±0.4
TG18	98.1±0.5	91.6±0.7	92.1±0.4

Based on three independent assays.

### *In vitro* Anti-bacterial Activity of HBD3 Transgenic Milk

The anti-bacterial activity of rHBD3 in the milk of transgenic goats, non-transgenic goats, and non-transgenic control milk containing HBD3 standard was evaluated by inhibition zone and liquid growth inhibition assays.

**Figure 5:**
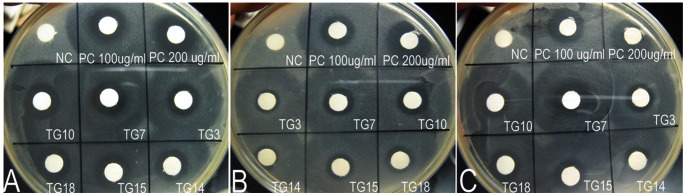
Anti-bacterial activity of rHBD3 in milk was measured by an inhibition zone assay. Bacterial suspensions *E. coli* (A), *S. aureus* (B) or *S. agalactiae* (C) were spread onto 100-mm agar dishes. Then, 20 µl milk samples were spotted on quantitative filter paper. The HBD3 standard was diluted in non-transgenic milk to 100 and 200 µg/ml. NC, milk samples from non-transgenic goats (negative control); PC, HBD3 standard (positive control); TG, milk samples from transgenic goats.

The anti-bacterial activity of HBD3 could be roughly compared by measuring the diameter of the clear zone (Fig. 5). Inhibition zones around filters containing 20 µl milk samples from six transgenic goats were clearly visible after incubation for 24 h, which were similar to those of non-transgenic control milk containing 100 µg/ml HBD3 standard. No inhibition zone formed around filters containing milk from a non-transgenic goat. This result demonstrated that milk samples from HBD3 transgenic goats showed an inhibitory activity against *E. coli*, *S. aureus* and *S. agalactiae,* and the anti-bacterial activity against *E. coli* was higher than that against *S. aureus* and *S. agalactiae*.

The MIC of rHBD3 and the HBD3 standard in milk against *E. coli*, *S. aureus* and *S. agalactiae* was measured by a liquid growth inhibition assay (Table 5). The results indicated that the MICs of rHBD3 in transgenic milk against *E. coli*, *S. aureus* and *S. agalactiae* were 9.5–10.5, 21.8–23.0 and 17.3–18.5 µg/ml, respectively. The MIC of rHBD3 in milk was similar to that of the HBD3 standard (*P>*0.05). These results were consistent with those obtained from the inhibition zone assay. Thus, the concentration of rHBD3 in transgenic milk is sufficient for inhibiting the growth of *E. coli, S. aureus* and *S. agalactiae*.

**Table 5:** The MIC of rHBD3 in the milk of transgenic goats.

Groups	The MIC of rHBD3 and HBD3 standards in milk (µg/ml)*		
	*E. coli*	*S. aureus*	*S. agalactiae*
HBD3 standard	9.5±0.3	21.5±0.6	18.0±0.4
TG3	10.3±0.3	21.8±0.5	18.3±1.0
TG7	10.0±0.4	22.8±0.5	17.3±0.9
TG10	9.5±0.5	22.8±0.8	18.0±0.7
TG14	10.0±0.4	23.0±0.4	18.3±0.6
TG15	10.5±0.3	22.8±0.5	17.8±0.9
TG18	9.5±0.3	23.0±0.4	18.5±0.6

* The minimal inhibitory concentration (MIC) is the lowest concentration of HBD3 at which 99.9% of the viable cells are inhibited. All assays were conducted in duplicate and repeated four times.

Within a column, No difference was found in MIC among the groups (*P>*0.05).

### *In vivo* Anti-bacterial Activity Assay

**Figure 6:**
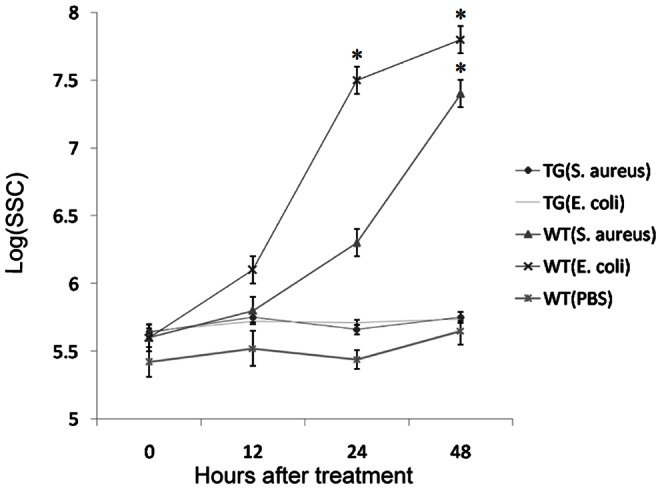
Somatic cell counts (SSC) in glands after infusion with *E. coli* or *S. aureus.* TG, transgenic goats (n = 6); WY, wild-type goats. Ten glands of wild-type goats were infused with *E. coli* or *S. aureus,* six glands of wild-type goats were infused with PBS. The data were represented as the mean±SEM, and analyzed by one-way ANOVA and least-significant difference tests. *P<0.05.

Mastitis resistance ability of HBD3 transgenic goats was investigated by intramammary infusion of viable bacterial inoculums (Table 6 and Fig. 6). Before treatment, the concentrations of rHBD3 in right and left glands of the six transgenic goats were 98.9±2.3 and 98.0±2.3 µg/ml, and no bacteria presented in the milk. The right and left glands of each transgenic (n = 6) and non-transgenic (n = 10) goats were infused with 1 ml *S. aureus* and *E. coli* inoculums (10^3^ CUF/ml in PBS), respectively. Six glands of three non-transgenic goats received 2 ml sterile PBS. Glands containing more than 10 CFU/ml were considered as infected. We observed that 9/10 and 8/10 glands of non-transgenic goats infused with *S. aureus* and *E. coli* were infected at 12 h after treatment, and the mean numbers of viable bacteria were 0.6×10^3^ and 1.1×10^3^ CFU/ml, and increased up to 2.9×10^3^ and 95.4×10^3^ CFU/ml at 48 h after infusion, respectively (Table 6). Furthermore, the mean SSC in infected glands significantly increased from7.2×10^5^ to 260.4×10^5^ and 12.4×10^5^ to 622.2×10^5^ cells/ml in *S. aureus* and *E. coli* infected goats, respectively (Fig. 6). In contrast, no bacteria presented in glands of transgenic goats and PBS-infused controls, and the SSC did not significantly change throughout the period with a mean value from 3.1×10^5^ to 5.8×10^5^ cells/ml.

**Table 6:** The colony forming unit in left and right glands post infusion with *S. aureus* and *E. coli*.

Groups	Concentration of rHBD3 before treatment	Glands treated	Glands infected*	The number of colony forming unit (×10^3^ CFU/ml)			
				0 h**	12 h	24 h	48 h
TG	98.9±2.3^a^	6(*S. aureus*)	0	0	0	0	0
TG	98.0±2.3^a^	6(*E. coli*)	0	0	0	0	0
WT	0	10(*S. aureus*)	9	0	0.6±0.1^a^	2.1±0.4^ab^	2.9±0.5^b^
WT	0	10(*E. coli*)	8	0	1.1±0.2^a^	7.6±0.9^a^	95.4±16.7^b^
WT	0	6(PBS)	0	0	0	0	0

* Glands containing fewer than 10 CFU/ml were considered as noninfected.

** Before treatment.

TG, transgenic goats; WT, wild-type goats.

a, b Within a row, values with different superscripts are significantly different from each other (*P*<0.05).

## Discussion

The production of transgenic dairy animals that express anti-bacterial proteins in their milk has been proposed as an alternative approach to enhance mastitis resistance [5]–[8]. In addition, the transgenic milk can be used as a source to purify anti-bacterial proteins for pharmacological and therapeutic applications. In this study, we generated a transgenic goat expressing rHBD3 in the mammary gland. The results indicated that the expression of rHBD3 in the lactating mammary gland efficiently inhibited the growth of bacteria that could cause mastitis.

Several investigations have reported that β-defensins play an important role in the response to mastitis, and protect the body from bacterial invasion [11], [12]. HBD3 has a broad-spectrum anti-bacterial activity against bacteria, fungi and enveloped viruses, and plays an important role in immunity [15]. Therefore, we hypothesized that exogenous expression of HBD3 in the mammary gland of dairy animals might enhance mastitis resistance. In this study, we examined the *in vitro* and *in vivo* anti-bacterial activity of rHBD3 in the milk of transgenic goats. The results indicated that rHBD3 in the milk of transgenic goats showed an efficient inhibitory activity against *E. coli, S. aureus* and *S. agalactiae in vitro*, and the anti-bacterial activity against *E. coli* was higher than those against *S. aureus* and *S. agalactiae*. The MICs of rHBD3 in transgenic milk against *E. coli, S. aureus* and *S. agalactiae* were similar to those of the HBD3 standard. In addition, the *in vivo* antibacterial activity assay has demonstrated that expression of rHBD3 (mean 98 µg/ml) in mammary gland of goats could protect against infection of *E. coli* and *S. aureus*. Previous studies have demonstrated that the MIC of HBD3 against *E. coli* and *S. aureus* are 6 and 12 µg/ml, respectively [13], [22], and the anti-bacterial and biochemical properties of recombinant and chemically synthesized HBD3 are indistinguishable from that of the isolated native peptide [13]. In contrast, the anti-bacterial activity of rHBD3 in this study was lower than those reported in previous reports. In this study, the anti-bacterial activity of rHBD3 and the HBD3 standards was measured in milk. Therefore, the reason for this discrepancy might be interference by the milk components.

Beta-casein is one of the most abundant proteins in milk. Therefore, the bovine β-casein promoter was used as the main control element to direct the expression of HBD3 in the mammary gland of transgenic goats. The mammary-specific expression vector has been assessed in mammary epithelial cells in a previous study [16]. In this study, transgenic fetal fibroblasts were used as donor cells for SCNT to produce transgenic goats. The expression level of rHBD3 in the mammary glands of transgenic goats cannot be determined before lactation. Furthermore, the transgenic technique is associated with random integration, and the positional effects may cause low expression of the transgene [23]. In the present study, two transgenic colonies with normal chromosomes and uniform small round cells were used as donor cells for SCNT, all live offspring were obtained from the transgenic cell line F1HBDC2. The concentrations of rHBD3 in milk ranged from 98 to 121 µg/ml at 15 days of lactation, and maintained at 90–111 µg/ml during the following 2 months. Although the concentration of rHBD3 in the milk of transgenic goats was not high, it was sufficient for inhibiting the growth of *E. coli, S. aureus* and *S. agalactiae*.

Transgenic donor cell preparation is an important step for SCNT to produce transgenic animals. In current study, seven transgenic clonal cell lines were obtained after the selection of G418 and confirmation by EGFP expression and PCR screening. After SCNT, we observed that the developmental rate of cloned embryos derived from various transgenic clonal cells was different, and this result is consistent with the finding reported in a previous publication [24]. The cloning efficiencies of transgenic fetal fibroblasts F1HBDC1 and F1HBDC2 were 0.4 and 1.6%, respectively, similar to those obtained by other research groups [25]–[27]. The offspring mortality was 46.2% (12/26), close to the data reported in previous publications [17], [27]. Although postnatal mortality was relatively high, seventy-eight percent (14/18) of the live born transgenic goats did not show abnormities. Composition analysis of the milk showed no significant difference in the content of total fat, protein, lactose, and dry matter between transgenic and non-transgenic goats. Moreover, the protein profile of the milk from transgenic and non-transgenic goats was similar. Expression of HBD3 in transgenic goats appeared to have no effect on the integrity of the mammary gland and milk production. This result is similar to the previous reports in bovine [6], [7]. Unlike several cationic peptides, HBD3 does not exhibit a cytotoxic activity against eukaryotic cells. It exhibits a very low hemolytic activity towards human erythrocytes when a high amount of the peptide (up to 500 µg/ml) is used [13], [28]. We inferred that the expression of HBD3 in transgenic goats would be safe for mammary epithelial cells. However, apart from the anti-bacterial activity, HBD3 has been reported to induce the secretion of IL-18, a proinflammatory cytokine in human keratinocytes [29]. The safety of constitutively expressed HBD3 in the mammary glands of transgenic goats needs to be studied in the future.

It has been reported that the expression of a number of anti-bacterial proteins occurs in response to mastitis, and that these proteins play an important role in the first line of defense that inhibits or delays bacterial infection of mammary tissue [1]. Clearly, these mechanisms are insufficient to prevent mastitis. Several transgenic animal models have been produced, which express human lysozyme [5], [6], [30], human lactoferrin [31], [32], bovine tracheal anti-bacterial peptide [33], and modified lysostaphin [7], [34]. Milk from transgenic mice containing 0.38 mg/ml recombinant human lysozyme was found to be bacteriostatic against *Pseudomonas fragi*, *Lactobacillus viscous* and a mastitis-causing strain of *S. aureus,* but not against a pathogenic strain of *E. coli.* [30]. Milk from human lysozyme transgenic goats (270±84 µg/ml) is capable of slowing the growth of mastitis-causing strains of *E. coli* and *S. aureus*, but does not affect the growth of an organism involved in cheese-making, *Lactococcus lacti* [5]. However, high concentrations of human lactoferrin (mean, 2.9 mg/ml) in the milk of transgenic cows does not protect them from infection by experimental *E. coli* [35]. Transgenic cows secreting lysostaphin at concentrations ranging from 0.9 to 14 µg/ml in their milk are resistant against *S. aureus* challenge *in vivo* [7], but lysostaphin is only active against S. aureus and it is thus not expected to be effective against non-staphylococcal infections. Our current study has demonstrated that rHBD3 in the milk of transgenic goats efficiently inhibits the growth of *E. coli* and *S. aureus* both *in vitro* and *in vivo.*

In conclusion, we successfully produced HBD3 transgenic goats by SCNT. The transgenic milk from these goats containing 90–121 µg/ml rHBD3 efficiently inhibited the growth of *E. coli, S. aureus* and *S. agalactiae*, which was similar to that of the HBD3 standards. The present study demonstrated that HBD3 could be an effective antibacterial protein to enhance the mastitis resistance of dairy animals.
